# Weight over-reporting is associated with low muscle mass among community-dwelling Japanese adults aged 40 years and older: a cross sectional study

**DOI:** 10.1186/s40101-022-00292-2

**Published:** 2022-05-05

**Authors:** Takashi Nakagata, Tsukasa Yoshida, Daiki Watanabe, Yukako Arishima-Hashii, Yosuke Yamada, Naomi Sawada, Hidekazu Shimada, Nobuo Nishi, Motohiko Miyachi

**Affiliations:** 1 National Institute of Health and Nutrition, National Institutes of Biomedical Innovation, Health and Nutrition, 1-23-1 Toyama, Tokyo 162-8636 Shinjuku-ku, Japan; 2 Sportology Center, Juntendo University Graduate School of Medicine, 2-1-1 Hongo, Bunkyo-ku, Tokyo 113-8421 Japan; 3 Institute for Active Health, Kyoto University of Advanced Science, 1-1 Nanjo Otani, Sogabe-cho, Kameoka-city, Kyoto Japan; 4 Faculty of Sport Sciences, Waseda University, 2-579-15 Mikajima, Tokorozawa, Saitama 359-1192 Japan; 5 Department of Health and Welfare (Current, Next Generation Development Department), Settsu City Local Government, 1-1-1 Mishima, Settsu-city, Osaka 566-8555 Japan; 6 Department of Public Health and Medical Affairs, Osaka Prefectural Government, 2-1-22 Otemae, Chuo-ku, Osaka-city, Osaka 540-8570 Japan

**Keywords:** Aging, Health checkup, Sarcopenia, Self-weighing, Weight perception

## Abstract

**Background:**

Weight misperception adversely affects health-related quality of life (HRQol); however, few studies have evaluated the relationship between weight misperception and muscle mass. This study aimed to examine the relationship of weight misperception with low muscle mass using skeletal muscle index (SMI) estimated by multifrequency bioelectrical impedance analysis (MF-BIA) among community-dwelling Japanese.

**Methods:**

Participants were 525 Japanese individuals aged 40–91 years old (male 89, female 436). Misperception was calculated by subtracting measured value from self-reported weight, presented as a percentage and categorized into tertiles based on sex (under-reporters, acceptable reporters, and over-reporters). Appendicular lean mass was estimated using MF-BIA, and low muscle mass was defined using SMI values of 7.0 and 5.7 kg/m^2^ for males and females, respectively, based on the Asian Working Group for Sarcopenia 2019 consensus. We evaluated the association between prevalence of low muscle mass and weight misperception (under-reporters and over-reporters) using multivariate logistic regression including covariate.

**Results:**

In total, 9.3% (49/525) of participants had low muscle mass. After adjusting for covariates, prevalence of low muscle mass was higher among over-reporters than acceptable-reporters (odds ratio [OR]; 2.37, 95% confidence interval [CI]; 1.03–5.44). Additionally, sensitivity analysis was performed on females, which confirmed that the prevalence of low muscle mass was higher in over-reporters than in acceptable-reporters (*OR*, 3.27; 95% *CI*, 1.18–9.12).

**Conclusion:**

Weight misperception was significantly correlated with low muscle mass, especially in over-reporters.

## Background

Misperceptions of height and weight have been defined as the difference between one’s self-perceived and objectively measured height and weight, respectively [1, 2]. Misperceptions of height and weight, which include under-reporting (objectively measured > self-reported) and over-reporting (objectively measured < self-reported), may be associated with variations in age, sex, body mass index (BMI), nationalities, ethnicity, and socio-economic status [3, 4]. According to substantial evidence, weight misperception has adverse effects on physical and psychological health [5, 6] and may negatively affect health outcomes. A previous study examined the association between weight misperception and health-related quality of life (HRQoL) among adolescents and adult populations. Weight misperception, in particular, the underestimation or over-reporting of weight, has been shown to be significantly associated with low HRQoL, after adjusting for the influences of age, chronic disease, and socioeconomic status [2]. Furthermore, weight misperception is related to more unhealthy and fewer healthy weight control behaviors [7].

Age-related loss of skeletal muscle mass and strength, which are associated with functional impairment and physical disabilities in elderly people [8], are key components of sarcopenia and frailty. As previously mentioned, various studies examined the association between weight misperception and health outcomes in a wide range of population [2, 5, 6]. However, few studies have examined the association between weight misperception and health outcomes related to skeletal muscle mass and strength. A previous study investigated the relationship of perceived weight status and muscle strength measured by hand grip strength conducted among 12,727 adults (mean age, 51.0 ± 16.6 years) using the Korea National Health and Nutrition Examination Survey (KNHANES) [9]. It demonstrated that under-reporters of weight status had higher odds of sarcopenia than accurate-reporters and over-reporters of weight status. Although mechanisms underlying the observed relationships between weight-related behaviors and hand grip strength are unclear, having correct perceptions of one’s own weight may not only be associated with HRQoL but also skeletal muscle mass.

Therefore, the present study aimed to examine the association between weight misperception and skeletal muscle mass, with the prevalence of low muscle mass measured using skeletal muscle index (SMI) values based on the Asian Working Group for Sarcopenia 2019 consensus.

## Methods

### Study design and participants

From July 1 to July 9, 2019, we conducted face-to-face surveys with 539 individuals (female = 447, male = 92), who were aged ≥ 40 years and resided in Settsu city, Osaka prefecture. The participants were recruited either when they participated in a specific health examination conducted at the Settsu Health Center or via Settsu’s public relations magazine and word of mouth. Details of study design and participants are described in our previous publication [10]. Of these participants, we excluded participants who lived outside Settsu ity (*n* = 10), missed skeletal muscle mass data (*n* = 2), refused to participate (*n* = 1), and were aged less than 40 (*n* = 1). Ultimately, this cross-sectional study included 525 adults (436 females and 89 males) aged 40 to 91. Before the study began, participants provided written consent to participate after receiving information about the procedures and purpose of the study. The study protocol was approved by the Ethics Review Board of the National Institute of Health and Nutrition (Ikikenhatsu 178-1). This study was carried out in accordance with the principles outlined in the Declaration of Helsinki and was registered with the Japan Clinical Trials Registry (UMIN000036880).

### Procedures

Each participant was asked to visit a public building in Settsu city once between 10 AM and 4 PM and complete questionnaires on daily life (e.g., about dwelling and physical activity) before collecting each one’s objective weight and height measurements. To obtain self-reported body weight and height measurements, participants were asked, “What is your current body weight and height?” in self-administered questionnaires. They filled out self-reported height and weight using 0.1 cm and 0.1 kg units, respectively. Other health-related variables, which include smoking habit, drinking habit, exercise habit, self-rated health, self-rated physical fitness, and self-rated economic condition, were assessed using a self-administered questionnaire. Details of these questionnaires are described in our previous study [10]. The heights of the participants were then objectively measured to the closest 0.1 cm using an analog height meter. Body weights were measured, and appendicular lean mass (ALM) was estimated using the multifrequency bioelectrical impedance analysis (MF-BIA) (MC-780A-N, TANITA, Tokyo, Japan), which were then validated using dual-energy X-ray absorptiometry (DXA) [11]. Participants were evaluated in their underwear and were asked to stand barefoot on toe-and-heel electrodes while holding handgrips, with their arms hanging down a few centimeters from the hips. The details of the MF-BIA device used were previously described. All the procedures were conducted in July 2019.

### Skeletal muscle mass measurements and definition of low muscle mass

We calculated the skeletal muscle index (SMI) as follows: *SMI* = ALM/height^2^. In this study, participants with lower SMI values for each sex were categorized as having “sarcopenia with low skeletal muscle mass.” According to the Asian Working Group for Sarcopenia, the cutoff SMI values using the bioelectrical impedance analysis (BIA) method were 7.0 and 5.7 kg/m^2^ in male and female, respectively [12].

### Weight misperception

Weight misperception was defined as the difference between self-reported and objectively measured values of weight and calculated as follows in our study. First, we calculated the difference of weight (kg) by subtracting objectively measured values from self-reported values of weight based on Ikeda’s study [4]. Next, to calculate the difference (%) against the objectively measured value, the difference obtained was then divided by the objectively measured value and multiplied by 100 (represented as %). For example, weight misperception of an individual with self-reported weight = 65.0 kg and objectively measured weight = 66.0 kg can be calculated as follows: −1.0 kg ÷ 66.0 kg × 100 % = −1.51%.

### Statistical analysis

For descriptive statistics, values of continuous variables were expressed as mean and standard deviation, or as median and interquartile range, while values of categorical variables were expressed as percentages (%). Continuous and ordinal participant characteristics were classified as follows: exercise habit (“Do you go walking or engage in other exercise at least once per week?” “yes” or “no”), smoking habit (“almost daily” or “sometimes” = yes, “used to, but quit,” or “never” = no), drinking habit (“almost daily,” or “sometimes,” = yes, and “almost never” or “never” = no); self-reported health (“very healthy” or “somewhat healthy” = good self-reported health, and “not very healthy” or “unhealthy” = poor self-reported health); self-reported physical fitness (“extremely confident” or “somewhat confident” = good self-reported physical fitness, and “slightly anxious” or “very anxious” = poor self-reported physical fitness); and self-reported socioeconomic status (“easy” or “somewhat easy” = high socioeconomic status, and “somewhat hard” or “hard” = low socioeconomic status). These variables were classified with reference to covariates used in our previous study [13], and these models were decided with reference to covariates used in previous studies, which examined the association between weight misperception on HRQoL [2].

Second, participants were first classified into sex-specific tertiles based on extent of weight misperception (under-reporters, acceptable reporters, and over-reporters) [14]. In order to evaluate the relationship between weight misperception and prevalence of low muscle mass, we used a logistic regression model to calculate the sex, age, and multivariable adjusted ORs, as well as 95% CI. In the logistic regression model, sex and age were inputted as covariates to calculate the adjusted ORs. To calculate the multivariable-adjusted ORs, continuous variables (i.e., sex, age, and BMI) were inputted into the logistic regression model as covariates (model 1). Then, exercise habit, smoking, drinking habit, self-reported health, self-reported physical fitness, and self-reported socioeconomic status were added to model 1 to calculate the multivariable-adjusted OR (model 2). Furthermore, linear regression analyses were conducted to obtain the correlation between the means of self-reported and objectively measured height, weight, and BMI. We used Microsoft Office Excel 2017 and PASW Statistics version 20.0 (SPSS, IBM Corp., Armonk, N.Y., USA) for data processing and statistical analyses, respectively. A two-tailed *p*-value lower than 5% was considered statistically significant.

## Results

Table 1 shows the characteristics of the participants including skeletal muscle mass and health-related information. In our study, the median weight misperception (interquartile range) was 0.9 (−0.3, 2.0)%, while the prevalence of low muscle mass using SMI was 9.3% (49/525).

**Table 1:** Characteristics of participants

Variables	All (*n* = 525)	Female (*n* = 436)	Male (*n* = 89)
Age, years	72 (67, 77)	72 (67, 77)	74 (69, 78)
Measured height, cm	154.3 ± 7.6	152.1 ± 5.8	164.9 ± 6.1
Measured weight, kg	53.7 ± 9.2	51.9 ± 8.3	62.3 ± 8.3
Measured BMI, kg/m^2^	22.5 ± 3.2	22.4 ± 3.3	22.9 ± 2.7
Weight misperception, kg^a^	0.5 (−0.2, 1.0)	0.5 (−0.1, 1.0)	0.3 (−0.6, 0.8)
Weight misperception, %^a^	0.9 (−0.3, 2.0)	1.0 (−0.1, 2.1)	0.5 −0.9, 1.3)
%fat, %	29.6 ± 7.9	31.2 ± 7.2	21.6 ± 5.8
ALM, kg	15.9 ± 3.2	14.9 ± 1.9	21.0 ± 3.3
SMI, kg/m^2^	6.6 ± 0.8	6.4 ± 0.5	7.7 ± 0.9
Low muscle mass, *n* (%)	49 (9.3%)	33 (7.6%)	16 (18.0%)
Exercise habit, *n* (%)			
Yes	396 (75.4%)	329 (75.5%)	67 (75.3%)
No	129 (24.6%)	107 (24.5%)	22 (24.7%)
Smoking habit, *n* (%)			
No	507 (96.6%)	429 (98.4%)	78 (87.6%)
Yes	18 (3.4%)	7 (1.6%)	11 (12.4%)
Drinking habit, *n* (%)			
No	337 (64.2%)	310 (71.1%)	27 (30.3%)
Yes	118 (22.5%)	126 (28.9%)	62 (69.7%)
Self-reported health, *n* (%)			
Good	440 (83.8%)	367 (84.2%)	73 (82.0%)
Poor	85 (16.2%)	69 (15.8%)	16 (18.0%)
Self-reported physical fitness, *n* (%)			
Good	269 (51.2%)	223 (51.1%)	46 (51.7%)
Poor	256 (48.8%)	213 (48.9%)	43 (48.3%)
Socioeconomic status, *n* (%)			
High	385 (73.5%)	324 (74.3%)	61 (69.3%)
Low	139 (26.5%)	112 (25.7%)	27 (30.7%)

Data are the means ± SD or percentages. Age and weight misperceptions are median values (interquartile ranges)

*BMI* Body mass index, *ALM* Appendicular lean mass, *SMI* Skeletal muscle index

^a^Calculated by subtracting measured values from self-reported values

**Fig. 1:**
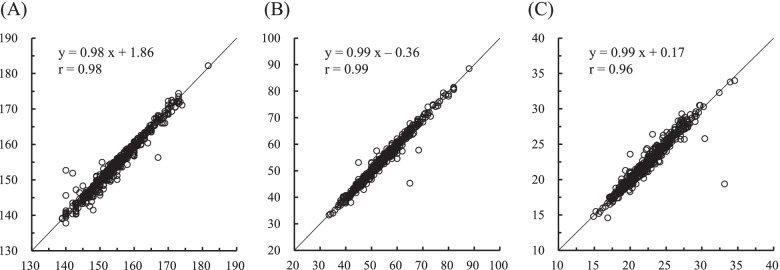
The scatter plots and linear relationship between the self-reported value and measured value. X-axis represents the self-reported value, and Y axis represents the measured value. The solid black line represents the identity line. **A** height, **B** weight, and **C** body mass index

Figure 1 shows the scatter plots and correlation between the means of self-reported and objectively measured height, weight, and BMI. All variables showed a strong positive correlation between self-reported and objectively measured characteristics (*r* = 0.96–0.99); however, there were some outliers from the identical line.

Table 2 shows the characteristics of the participants with weight misperceptions. Participants were divided into three groups based on sex-specific weight misperception tertiles: < 0.4%, 0.4–1.7%, and ≥ 1.7% in female and < −0.4%, −0.3–1.1%, and ≥ 1.1% in male, respectively. The median difference between self-reported weight and objectively-measured weight among under-reporters, accurate-reporters, and over-reporters were −0.8, 0.9, and 2.4%, respectively. We found no significant difference in age and height among under-reporters, accurate-reporters, and over-reporters. Results show that over-reporters have lower body weight, BMI, and SMI.

**Table 2:** Physical characteristics by the results of weight misperception

Variables	Weight misperception		
	Under-reporters	acceptable-reporters	Over-reporters
Number (female/male)	174 (145/29)	176 (146/30)	175 (145/30)
Age, yrs	72 (67, 77)	72 (67, 76)	74 (68, 78)
Height, cm	154.5 ± 7.9	154.4 ± 7.3	154.0 ± 7.6
Weight, kg	56.2 ± 9.4	53.4 ± 8.7	51.4 ± 8.9
BMI, kg/m^2^	23.5 ± 3.4	22.3 ± 2.9	21.6 ± 3.0
%fat, %	31.5 ± 8.3	28.9 ± 7.7	28.3 ± 7.3
ALM, kg	16.2 ± 3.0	16.1 ± 3.2	15.5 ± 3.2
SMI, kg/m^2^	6.7 ± 0.7	6.7 ± 0.8	6.5 ± 0.8
Low muscle mass, *n* (%)	8 (4.6%)	12 (6.8%)	29 (16.6%)
Exercise habit, *n* (%)			
Yes	124 (71.3%)	134 (76.1%)	138 (78.9%)
No	50 (28.7%)	42 (23.9%)	37 (21.2%)
Smoking habit, *n* (%)			
No	166 (95.4%)	172 (97.7%)	169 (96.6%)
Yes	8 (4.6%)	4 (2.3%)	6 (3.4%)
Drinking habit, *n* (%)			
No	107 (61.5%)	119 (67.6%)	111 (63.4%)
Yes	67 (38.5%)	57 (32.4%)	64 (36.6%)
Self-reported health, *n* (%)			
Good	149 (85.6%)	143 (81.3%)	148 (84.6%)
Poor	25 (14.4%)	33 (18.8%)	27 (15.4%)
Self-reported physical fitness, *n* (%)			
Good	93 (53.4%)	87 (49.4%)	89 (50.9%)
Poor	81 (46.6%)	89 (50.6%)	86 (49.1%)
Socioeconomic status, *n* (%)			
High	121 (69.5%)	135 (76.7%)	129 (73.7%)
Low	52 (29.9%)	41 (23.3%)	46 (26.3%)

Data are the means ± SD or percentages. Weight misperception and age are median (interquartile range). Sex-specific weight misperception tertiles in female: under-reporters, < 0.4 (%); acceptable-reporters, 0.4–1.7 (%); over-reporters, ≥ 1.7 (%). Sex-specific weight misperception tertiles in male: under-reporters, <−0.4 (%); acceptable-reporters, 0.3–1.1 (%); over-reporters, ≥ 1.1 (%)

*BMI* Body mass index, *ALM* Appendicular lean mass, *SMI* Skeletal muscle index

Table 3 shows the multivariate-adjusted ORs of the prevalence of low muscle mass by tertiles of weight misperception. After adjusting for sex, age, and BMI (model 1), the OR for the prevalence of low muscle mass was higher (2.01 [95% *CI*, 0.90–4.47]) in the over-reporting group than in the acceptable-reporting group. After adjusting for model 1 and other variables (model 2), the OR was higher (2.37 [95% *CI*, 1.03–5.44]) in the over-reporting group than in the acceptable-reporting group. A total of 436 females were included in the sensitivity analysis after excluding males because of low statistical power. The results of this analysis confirmed the results from the main analysis (3.27 [95% *CI*, 1.18–9.12], Table 4).

**Table 3:** Multivariate-adjusted odds ratios and 95% confidence intervals for prevalence of low muscle mass by the results of weight misperception

	*n*	Number of low muscle mass	*OR* (95% *CI*)	Model 1 *OR* (95% *CI*)	Model 2 *OR* (95% *CI*)
Under-reporters	174	8 (4.6%)	0.66 (0.26–1.65)	0.76 (0.27–2.11)	0.97 (0.34–2.86)
Acceptable-reporters	176	12 (6.8%)	1.00 (reference)	1.00 (reference)	1.00 (reference)
Over-reporters	175	29 (16.6%)	2.72 (1.34–5.52)	2.01 (0.90–4.47)	2.37 (1.03–5.44)

Model 1, adjusted for sex, age, and BMI. Model 2, model 1 plus exercise habit, smoking habit, drinking habit, self-reported physical fitness, self-reported health, and self-reported socioeconomic status

*CI* Confidence interval, *OR* Odds ratio

**Table 4:** Multivariate-adjusted odds ratios and 95% confidence intervals for prevalence of low muscle mass by the results of weight misperception in female

	*n*	Number of low muscle mass	*OR* (95% *CI*)	Model 1 *OR* (95% *CI*)	Model 2 *OR* (95% *CI*)
Under-reporters	145	4 (2.8%)	0.56 (0.16–1.97)	0.84 (0.22–3.18)	1.25 (0.30–5.16)
Acceptable reporters	146	7 (4.8%)	1.00 (reference)	1.00 (reference)	1.00 (reference)
Over-reporters	145	22 (15.2%)	3.55 (1.47–8.60)	2.61 (1.00–6.81)	3.27 (1.18–9.12)

Model 1, adjusted for age and BMI. Model 2, model 1 plus exercise habit, smoking habit, drinking habit, self-reported physical fitness, self-reported health, and self-reported socioeconomic status

*CI* Confidence interval, *OR* Odds ratio

## Discussion

To our knowledge, this study is the first to evaluate the relationship between weight misperception and low muscle mass using MF-BIA in community-dwelling Japanese adults. Using MF-BIA-measured SMI, the present study indicates that participants who over-report body weight—self-reported weight was larger than objectively measured weight (median 0.9 %, [interquartile range (−0.3, 2.0)])—have higher odds of low muscle mass, as compared to the acceptable-reporting group (odds; 2.37 [95% *CI*, 1.03–5.44]).

Self-reported height and weight are often used for convenience in annual health checkups and large population surveys [15, 16], and previous studies have shown a high correlation between measured and self-reported height and weight among Japanese adults [4, 17–19]. Previous studies have been conducted focusing on the difference between measured and self-reported values and considered the associations among misperceptions of being “fat,” HRQoL, and unhealthy weight control behaviors [2, 5–7]. For example, studies among Korean subjects who misperceived their weight were associated with significantly impaired HRQoL and sarcopenia based on hand grip strength [2, 9]. Weight misperception is also possibly associated with skeletal muscle mass; however, to the best of our knowledge, no relevant studies have been conducted. In our logistic analysis, misperception of body weight was significantly associated with low muscle mass using SMI; over-reporting group had higher ORs for the prevalence of low muscle mass than the acceptable-reporting group (2.37 [95% *CI*, 1.03–5.44], Table 3). In addition, sensitivity analysis indicated that excluding males did not change that finding; the odds of low muscle mass in over-reporting groups was significantly higher than in acceptable-reporters (3.27 [95% *CI*, 1.18–9.12], Table 4).

Although the mechanism underlying misperception of weight-related low muscle mass is unclear in our cross-sectional study, the possible factors for misperceiving body weight and their relationship to health outcomes have been discussed previously. One potential factor could be body status; BMI among over-reporters was lower than among acceptable and under-reporters in this study (under-reporters, 23.5 ± 3.4; acc-reporters, 22.3 ± 2.9; and over-reports, 21.6 ± 3.0). According to Ikeda’s study on the characteristics of weight misperception among the general adult population in Japan based on the National Household Surveys, 1986, overweight and obese participants (BMI ≥ 25), both male and female, significantly under-reported their body weight as compared to their measured weight (range −5.9 kg to −1.3 kg) [4]. In contrast, underweight participants (BMI < 18.5) in that study over-reported their body weight (mean values, male; 2.4 kg, female; 1.1 kg) [4]. Previous studies showed that low BMI was associated with the prevalence of low muscle mass among Japanese populations [10, 20], and the association between over-reporting body weight and low BMI might be related to low muscle mass in our study. Furthermore, low BMI may be associated with lower total energy intake per day. Body status has been linked to misreporting energy intake [21, 22], while being underweight (*BMI* < 18.5 kg/m^2^) has been associated with over-reporting energy intake based on the ratio of energy intake per day to estimated energy requirement in US adults using data from the National Health and Nutrition Examination Survey (NHANES) 2003–2012 [14]. Taking information from these previous studies [14, 21, 22], weight over-reporting may be associated with low muscle mass. Further studies are still needed to examine the relationship between weight misperception and energy intake. Second, individuals who misperceive their own body weight suggest that they may not have been weighed recently. Our study did not ask participants about whether they had concrete knowledge of their weight (e.g., do they own a scale, have a habit of self-weighing, or if they had been weighed at the doctor recently), although previous studies investigate the association between self-weighing frequency and healthy weigh-related behaviors. Houston et al. have examined the relationship of self-weighing frequency among the 4 quartiles (never, less than one time a week, one time a week, and several times a week or more) with BMI, physical activity, and other health-related measures among US adults and found that frequent self-weighing was associated with less sedentary time, more vigorous physical activity, and several healthier practices, including nutrition and healthy dietary habits in a cross-sectional study [23]. Furthermore, Zheng et al. examined the temporal patterns of self-weighing behavior and weight changes in free living over 52 weeks; participants who consistently self-weighed more than 6 days/week achieved higher weight loss compared to other participants [24]. Self-weighing or self-monitoring of weight, which are often recommended to prevent weight gain, to lose weight, or prevent weight regain [16, 25], could help increase their awareness of healthy weigh-related behaviors and may be linked to low muscle mass indirectly.

However, our study has several limitations. First, this was a cross-sectional study; therefore, further cohort studies are warranted to establish a relationship between low muscle mass and weight misperception. Second, the participants were not randomly selected from the city in our study; they may have been more health conscious than the general population, suggesting that selection bias might have been present. In our previous study, face-to-face participants had lower percentages of being an alcohol drinker or current smoker and a higher level of self-reported socioeconomic status than participants recruited by stratified random sampling in Settsu city [10]. Therefore, further research should be conducted with randomized sampling. Third, the sample size, especially for males, was small (*n* = 89); therefore, further studies with a larger sample size are required to confirm our data. Fourth, we investigated self-reported height and weight only once; therefore, we could not describe the reproducibility of weight misperception in our participants. Thus, further studies are needed to evaluate the reproducibility of the self-reported values. Finally, we did not measure muscle strength (e.g., handgrip strength) and gait speed; for screening and diagnosis of sarcopenia, both measurements were included.

## Conclusion

This study showed that individuals who over-report their body weight have higher odds of low muscle mass among community-dwelling Japanese adults aged 40 years and older. Our findings suggest that public health strategies should not only promote healthy weight but also focus on fostering accurate weight perception among the population.

## Data Availability

The datasets used and/or analyzed during the study are available from the corresponding author on reasonable request.

## Abbreviations

SMI: Skeletal muscle index
ALM: Appendicular lean mass
BMI: Body mass index
CI: Confidence interval
OR: Odds ratio
DXA: Dual-energy X-ray absorptiometry
